# Complete genome sequence of *Franconibacter pulveris* SL.qac10, a putative QAC-resistant isolate from an indoor built environment

**DOI:** 10.1128/mra.01096-24

**Published:** 2024-11-27

**Authors:** S. Liu, K. M. Leung, G. K. K. Lai, S. D. J. Griffin

**Affiliations:** 1 Shuyuan Molecular Biology Laboratory, The Independent Schools Foundation Academy, Hong Kong, China; University of Southern California, Los Angeles, California, USA

**Keywords:** *Franconibacter pulveris*, QAC, quaternary ammonium compound, disinfectant, co-resistance, AMR

## Abstract

*Franconibacter pulveris* SL.qac10 was isolated at a school in Hong Kong from swabs of flooring frequently cleaned with a quaternary ammonium compound (QAC)-based disinfectant. Its complete genome, a single chromosome and circular plasmid totalling 4.88 Mbp (56.50% G + C), was established by hybrid assembly.

## ANNOUNCEMENT

Formerly classified as *Enterobacter pulveris* and *Cronobacter pulveris* (1), *Franconibacter pulveris* is a Gram-negative, motile, rod-shaped facultative anaerobe initially found in dried fruit powder (2) and powdered infant formula (3). Some industrial potential is claimed for strains found to resist high temperatures and pressures (4) and to degrade hydrocarbons (5). Here, *F. pulveris* SL.qac10 was isolated as part of a broader study of quaternary ammonium compound (QAC) and antimicrobial co-resistance in a built environment.

A moistened cotton bud was used to swab flooring in a common area of a school frequently cleaned with a QAC-based disinfectant (22.264658N 114.130000E; Telegraph Bay, Hong Kong). After rinsing with 1 mL 0.9% (wt/vol) saline, 100 µL of this extract was used to inoculate 1.5 mL 6.5% NaCl Luria broth (6) (a high-salt medium since QAC-resistant isolates are often osmotolerant (7)). Following overnight incubation, a 100 µL aliquot of the broth was spread onto Luria agar containing 0.02% (wt/vol) benzalkonium chloride. Resultant colonies were transferred to Luria agar containing 0.075% benzalkonium chloride, and SL.qac10 was found to grow readily. MIC test strips (Liofilchem, Italy), used according to the manufacturer’s instructions, showed SL.qac10 resistant to fosfomycin (>256 mg/L) and to ampicillin (>48 mg/L) after overnight growth. For ampicillin, heteroresistance was indicated by colony growth within the initial clear zone after 48 h (8, 9). SL.qac10 was passaged to purity on Luria agar and a single colony streaked onto this medium for overnight growth before harvesting for DNA extraction (DNeasy Powersoil Pro Kit, Qiagen GmbH). All agar/broth incubations were at 27°C.

Paired-end short-read libraries (NEBNext Ultra DNA Library Prep Kit, Illumina, Inc.) were sequenced via the NovaSeq 6000 platform using an SP PE250 Flow Cell and v1.5 Reagent Kit. A total of 5,007,846 raw reads (mean length: 250 bp) (SRX26089772) quality-filtered and trimmed using TrimGalore! v0.6.7 (stringency:3; -e:0.2) produced 4,999,136 read pairs (mean length: 247 bp, total: ~1.24 Gbp). Long-read libraries prepared from the same extracted DNA using Rapid Barcoding Kit SQK-RBK004 without size selection were sequenced via Oxford Nanopore’s Spot-ON Flow Cell (vR9.4.1), MinION sequencer, and MinKNOW v20.06.2 software, with base-calling by Guppy v6.1.5. The final long-read data set of 247,869 raw reads (mean length: 9,468 bp) (SRX26089773) was trimmed by Filtlong v0.2.1 (min_length: 2000; target_bases: 500 Mbp; length_weight: 10), selecting 82,241 reads (mean length: 15,864 bp, N50: 19,187, total: ~1.50 Gbp) to scaffold the assembly. Default parameters were used for all software, unless otherwise specified.

Illumina and MinION data sets were combined using Unicycler v0.5.0 (10) yielding a single chromosome (4,803,736 bp, 56.51% G + C; rotated to begin *dnaA*) and a circular plasmid (76,403 bp, 55.44% G + C; not rotated) with mean coverage 284× (judged 100% complete with 0.62% contamination by CheckM v1.2.3 [11]), which were submitted to NCBI PGAP v6.8 (12) for annotation. SL.qac10 shares an average nucleotide identity (ANIb by BLAST+ 2.11.0 [13]) of 98.96% with *Franconibacter pulveris* DJ34 (GCA_001077855.1).

PubMLST (14) found alignment in plasmid pSL.qac10 to IncFIA allele 12 (*repE*). Table 1 lists the antimicrobial/disinfectant-resistance genes predicted by RGI v6.0.3 (15) and by PGAP v6.8, all chromosomally encoded.

**TABLE 1:** Antimicrobial and disinfectant resistance genes predicted by RGI v6.0.3 (strict hits) (15) and by PGAP v6.8 (12)^*a*^

Locus	Gene by PGAP v6.8	Gene by RGI v6.0.3	Antimicrobial resistance	Disinfectant resistance	Resistance mechanism^*b*^
ACBV55_10425	*fos*	*fosA8*	✓		I
ACBV55_04495	*bla*	MAL-2^*c*^	✓		I
ACBV55_05385 to ACBV55_05390	*acrAB*	*acrA-adeF*	✓	✓	E
ACBV55_07250	*msbA*	*msbA*	✓		E
ACBV55_10605		*qacG*		✓	E
ACBV55_11380	*marR*	*marR*	✓	✓	T, E
ACBV55_11385	*marA*	*marA*	✓	✓	E
ACBV55_11860 to ACBV55_11865	*mdtJI*	*kpnEF*	✓	✓	E
ACBV55_15280	*baeR*	*baeR*	✓		E
ACBV55_16940	*acrD*	*adeF*	✓		E
ACBV55_18185 to ACBV55_18195	*mprA-emrAB*	*emrRAB*	✓		E
ACBV55_18250	*csrA*	*rsmA*	✓		E
ACBV55_20985	*crp*	*crp*	✓		E
ACBV55_01115	*tuf*	*tuf*	✓		T
ACBV55_03395	*ftsI*	*pbp3*	✓		T
ACBV55_15885	*glpT*	*glpT*	✓		T
ACBV55_20875	*tuf*	*tuf*	✓		T

^*a*^ All are chromosomally encoded.

^*b*^ Resistance mechanisms: antibiotic inactivation (**I**); antibiotic efflux (**E**); and antibiotic target alteration (**T**).

^*c*^ Loose fit by CARD RGI v6.0.3 to *bla*(MAL-2).

## Data Availability

Assembly data for Franconibacter pulveris SL.qac10 are available through NCBI under BioProject PRJNA1148304, with GenBank accessions CP167165-CP167166.
